# Development and characterization of DNA aptamer against Retinoblastoma by Cell-SELEX

**DOI:** 10.1038/s41598-022-20660-3

**Published:** 2022-09-28

**Authors:** Bhavani Shankar Maradani, Sowmya Parameswaran, Krishnakumar Subramanian

**Affiliations:** 1 L&T Ocular Pathology Department, Vision Research Foundation, Sankara Nethralaya, No. 41, College road, Nungambakkam, Chennai, 600006 Tamil Nadu India; 2 Radheshyam Kanoi Stem Cell Laboratory, Vision Research Foundation, Chennai, India; 3 School of Chemical and Biotechnology, SASTRA Deemed-to-Be University, Thanjavur, India

**Keywords:** Paediatric cancer, Eye cancer, Diagnosis

## Abstract

Retinoblastoma (RB) is the most common paediatric intraocular tumour. The management of RB has improved the survival and vision with recent advances in the treatment. Improved therapeutic approaches focussing on targeting tumours and minimizing the treatment-associated side effects are being developed. In this study, we generated a ssDNA aptamer against RB by cell-SELEX and high-throughput sequencing using Weri-RB1 cell line as the target, and Muller glial cell line Mio-M1 as the control. Three aptamers were selected based on the number of repetitions in NGS and phylogenetic relationship and evaluated by flow cytometry to assess their binding affinity and selectivity. The dissociation constant, Kd values of three selected aptamers were found to be in the nanomolar range. Aptamer VRF-CSRB-01 with the best binding affinity and a Kd value of 49.41 ± 7.87 nM was further characterized. The proteinase and temperature treatment indicated that VRF-CSRB-01 targets surface proteins, and has a good binding affinity and excellent selectivity under physiological conditions. The aptamer VRF-CSRB-01 was stable over 72 h in serum and 96 h in cerebral spinal fluid and vitreous. With the high affinity, specificity, stability and specific recognition of clinical RB tumours, VRF-CSRB-01 aptamer holds potential for application in diagnosis and targeting RB.

## Introduction

Retinoblastoma (RB) is the most common primary intraocular malignant tumour in children, with an incidence of 1:16,000 to 18,000 live births worldwide^1^. Retinoblastoma is highly aggressive and leads to intra-orbital, intracranial, and even systemic metastasis. However, advances in imaging, local forms of therapy, and systemic chemotherapy have improved outcomes in better survival and vision salvage. Recent advances in histopathology reporting^2^, tumor sub-type identification^3,4^ and cell-free DNA diagnostics^5,6^ have improved the prognosis of RB. Similarly, researchers have been focusing on newer modes of therapy such as intensity modulated radiation therapy^7^, proton therapy^8^, and oncolytic virus therapy^9^. The treatment strategies are being developed to selectively target the tumour cells with less damage to the normal cells, thereby reducing the treatment-related additional risks.

Aptamers were previously used in ophthalmic care^10^, but they have recently sparked interest for imaging^11–13^ and drug delivery^14–16^. Aptamers can serve as promising agents to target a wide range of molecules for diagnosis and therapeutic purposes as they show high affinity and specificity for their targets^17^. Aptamers can be DNA or RNA molecules and are single-stranded oligonucleotides selected from a library containing random sequences using a method called Systematic Evolution of Ligands by Exponential Enrichment (SELEX). Aptamers exhibit several advantages like low molecular weight, thermal stability, non-toxicity, lack of immunogenicity, cost-effective synthesis, and controllable post-production modifications, making aptamers an alternative molecular probe in clinical, biomedical, and therapeutic applications^18–20^. Aptamers with high binding affinity could be generated with the application of the latest technologies in aptamer development, such as Capillary Electrophoresis SELEX^21,22^, microfluidics based Magnetic SELEX^23^, Cell-SELEX^24–26^, High throughput SELEX^26–29^, and In-vivo SELEX^30,31^ etc.

Aptamers designed against specific proteins like EpCAM, Nulceolin, HMGA-2, ABCG2, CD133, and CD44, etc. which are highly expressed in several cancers, are used in targeting RB by different strategies like conjugation with chemotherapeutic drugs like Doxorubicin, siRNA, miRNA, and pri miRNA, etc.^32–35^. There are a few challenges and limitations with the application of these aptamers in RB. The high-level over-expression of EpCAM in several cancers^36^ led to the use of it as a therapeutic target and marker^37^. The EpCAM ectodomain is shed for activation, and probes that target the extracellular domain in cell surface molecules cannot target tumour cells^38,39^. The main limitation with the NCL and HMGA2 is that they are nuclear proteins, which need vehicles for entry into tumor cells. NCL also shuttles from the nucleolus to the nucleoplasm, cytoplasm, and cell surface, with varying cell surface expression^40^. AS1411 aptamer targeting NCL has delivered drugs to other normal organs such as the heart and brain^41^. ABCG2, CD133, and CD44 are cancer stem cell markers^42^, and the cancer stem cells account for 0.05–1% in entire tumor population^43^. Furthermore, these aptamers are created using recombinant proteins that have different conformations than native proteins, resulting in varying binding affinity to target proteins expressed on RB cells. Due to complexity of tumor heterogeneity in Retinoblastoma^44^, it remains highly challenging to effectively utilize the above targets.

Cell-SELEX is a modification of traditional SELEX, wherein live cells are used as targets when compared to a specific target ligand. Due to oncogenic mutations, the cell surface molecules are either overexpressed or altered, which can be targeted to develop cancer-specific aptamers by cell-SELEX technology. Cell-based SELEX has an advantage over protein SELEX because this approach targets proteins expressed on cell surfaces^45^, and the developed aptamers will be able to bind to the cell-surface molecules in their native conformation^46^. Cell-SELEX does not require any prior knowledge of the molecular target expressed on the cells, allowing for the generation of aptamers bound to various targets. Cell-SELEX helps to discover new biomarkers of unknown molecular events and signatures specific to cell of interest^47^. The application of cell-based SELEX is more desirable for the selection of aptamers to target cancer cells, which shows great potential in biomedical research and the development of tumour-specific diagnosis and therapeutics^48^.

In this study, we combined cell-SELEX with next-generation sequencing to select ssDNA aptamer sequences that specifically bound to the Retinoblastoma. A Retinoblastoma cell line, Weri-RB1, was used as the target, and a Muller Glial cell line was used as a negative control to generate RB-specific aptamers. Flow cytometry was used to evaluate the enrichment of the selection process and the binding affinity and selectivity of selected aptamers. Moreover, the translational applicability of the developed aptamer was assessed using tumour tissue sections. The results indicate that the obtained aptamer can specifically recognize RB cells and holds great potential to be used as a molecular recognition probe for detection and therapeutic applications in Retinoblastoma.

## Results

### Selection and enrichment of aptamers against Weri-RB1 cells through cell‑SELEX

**Figure 1:**
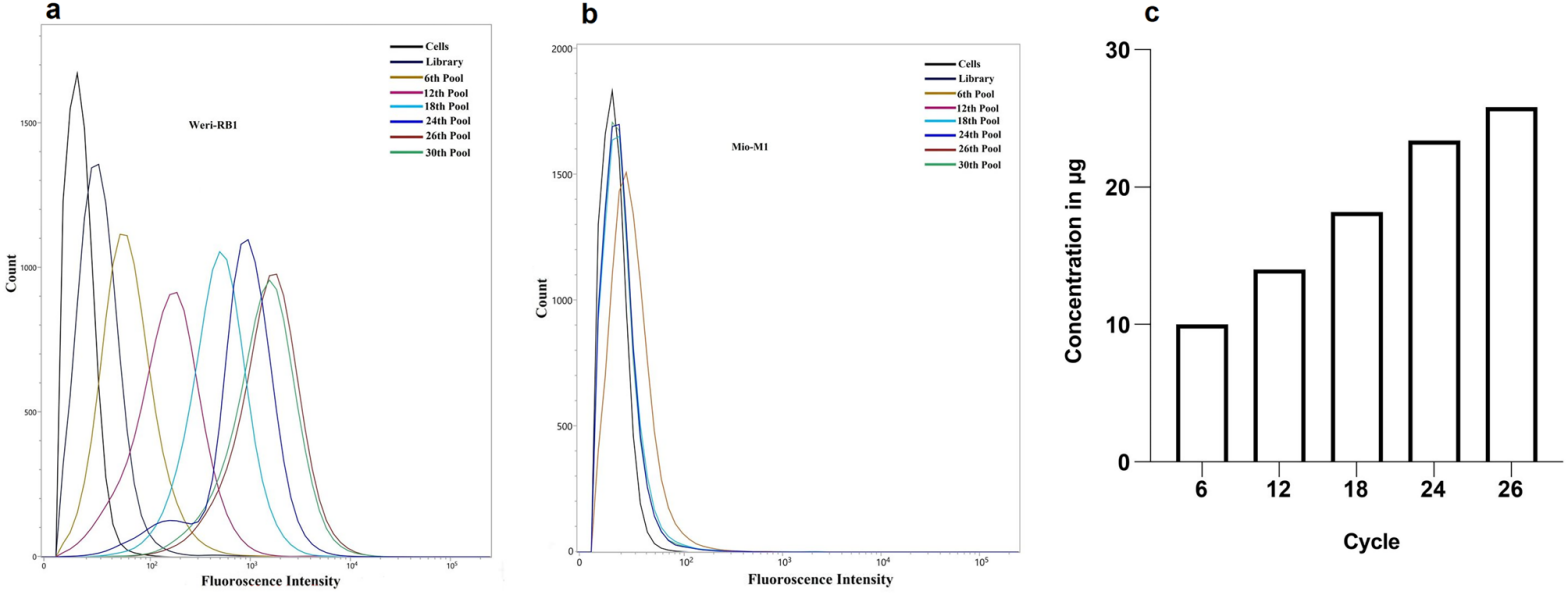
Monitoring the enrichment of the selected DNA libraries regarding the target cell line Weri-RB1 and control cell line Mio-M1 used for counter selection steps during the cell-SELEX process. (**a**) Fluorescence intensities of target cells incubated with FITC-labelled ssDNA pools from the initial library to the thirty-selection cycle. (**b**) Fluorescence intensities of negative control cells incubated with FITC-labelled ssDNA pools from the initial library to the thirty-selection cycle. (**c**) Represented is the ssDNA amount bound to the Weri-RB1 cells from the sixth to the twenty sixth selection cycle.

To obtain DNA aptamers specific for Retinoblastoma (RB), we employed a Cell-SELEX approach using the RB cell line, Weri-RB1, as the target cells. The first four rounds of positive selection were performed to enrich the sequences specific to Weri-RB1 cells, which are present in low copy numbers in the initial library pool. Starting from the fifth SELEX round the positive selection was preceded by counter-selection steps against the Mio-M1 cells. During each round of selection, PCR amplified dsDNA was identified as a single band with a molecular weight of 84 bp (data not shown). Affinity purification on streptavidin-sepharose beads followed by alkaline separation was performed to get FITC-labelled ssDNA, which was used as an input for the next round of selection. The initial ssDNA library, 6, 12, 18, 24, 26, 28, and 30th round pools were tested for selection enrichment. As shown in Fig. 1a,b with the progression of selection cycles, there was a steady increase in the fluorescence intensity on the Weri-RB1 cells till the 26th cycle, whereas there was no obvious change in fluorescence intensity on the Mio-M1 cells.

**Figure 2:**
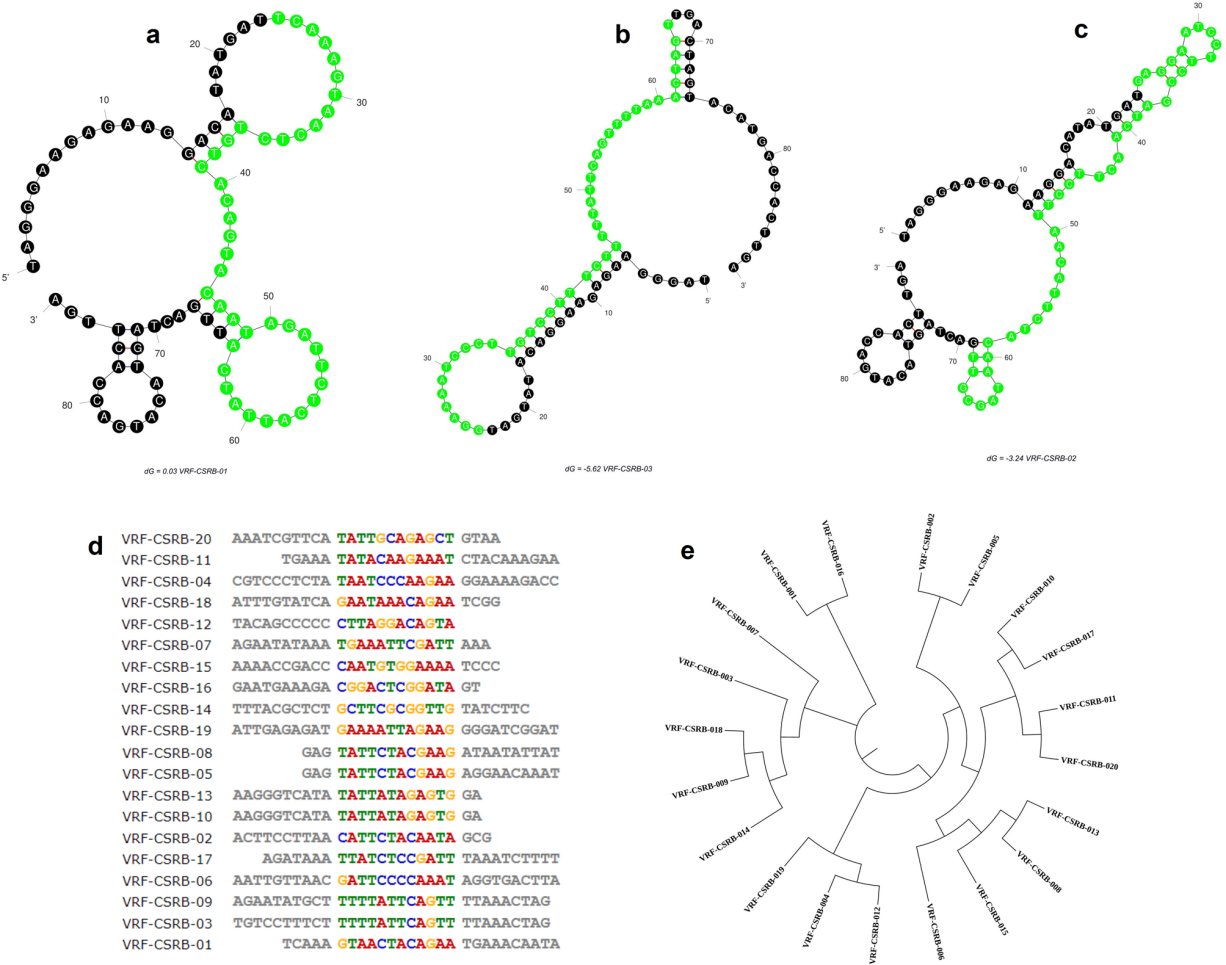
Secondary structure, sequence homology and phylogenetic tree. (**a**–**c**) Predicted secondary structures for three aptamer candidates, VRF-CSRB-01, VRF-CSRB-02 and VRF-CSRB-03 selected for further. The presented predicted secondary structures were the ones with lowest ΔG. Constant sequence regions are highlighted in black, and green represents the random regions. (**d**) Sequence homology analysis by MEME online software. (**e**) Phylogenetic analysis of aptamer candidate sequences was performed using the Tree Builder function in Geneious software (Geneious version 9.1.4, https://www.geneious.com).

The progression of the enrichment was also monitored by measuring the amount of ssDNA bound to target cells, obtained after PCR and ssDNA generation (Fig. 1c). The amount of DNA bound to target cells with the progression of the SELEX cycles further confirmed the enrichment. Hence, the enriched pools were sequenced using Illumina sequencing by Base-pair Biotechnologies, Texas (US). Based on the abundance in the sequenced pools and homology within their variable core region (Fig S1, S2 and Table S1, S2), the top 20 most frequent oligonucleotide sequences were identified. Secondary structure analysis of these sequences was done with the primer and random regions to determine the folding characteristics of the aptamers (Fig S3). VRF-CSRB-19 has a single stem-loop (SL) structure, VRF-CSRB-01 (Fig. 2a), 06, 09 and 12 show three SLs, VRF-CSRB-02 (Fig. 2b), 04, 07, and 10 have complex SLs with normal SLs, and VRF-CSRB-03 (Fig. 2c), 05, 08, 11, 14, 15, and 18 show two SL structures. Moreover, VRF-CSRB-13 has a complex SL. The nucleotides involved in the random regions were investigated to find out the possible binding motifs. There were no conserved motifs across the aptamer’s candidates selected. Based on MEME homology, three possible motifs, one with 12 bases, one with 7 and the last with 6 bases, were identified. We investigated the conserved nucleotides of the motifs identified in the SL of secondary structures of the aptamers (Fig. 2d). Within the 12 bases motif, the conserved sequence ‘TATT’ was found in VRF-CSRB-03, 05, 08,09, 13, 16, 17 and 20; ‘GAAA’ in VRF-CSRB-07, 11, 15 and 19. According to their free energy, VRF-CSRB-03 has the lowest free energy (ΔG = -5.62 kcal/mol) and VRF-CSRB-19 has the highest one (ΔG = 0.29 kcal/mol). The secondary structure with lower free energy, is the most stable one. Multiple sequence alignments of the aptamer sequences showed no conserved motifs or consensus sequences, confirming MEME results. However, the phylogenetic analysis revealed distinct evolutionary tree (Fig. 2e). Based on the phylogenetic relationship and sequence alignment, three main families of aptamers were identified, meaning that they do not share a similar sequence for conserved motifs or motifs for conserved sequence. VRF-CSRB-01 and VRF-CSRB-16 are members of the same family; VRF-CSRB-03, VRF-CSRB-07, VRF-CSRB-09, VRF-CSRB-14, VRF-CSRB-18 are members of the same family; and VRF-CSRB-02, VRF-CSRB-05, VRF-CSRB-06, VRF-CSRB-20 are members of the same family. Among the 20 selected aptamer sequences, three candidate aptamers (VRF-CSRB-01, VRF-CSRB-02, and VRF-CSRB-03) from different families were chosen based on the highest number of repeats in the respective family, and the percentage of abundance in the sequenced pools (Fig S4). Then, the selected aptamers were synthesized with FITC labels for characterization (Table 1).

**Table 1:** Oligonucleotide sequences of selected aptamer candidates. Top three from most frequent oligonucleotide sequences obtained after next-generation sequencing (NGS) of the 26th Cell-Selex cycle. Only the sequence corresponding to the random region is shown.

Aptamer name	Sequence in 5’ to 3’
VRF-CSRB-01	GGAAAATCCCTTGTCCTTTCTTTTTATTCAGTTTTAAACTAG
VRF-CSRB-02	AGAATATGCTAAAAAAAACCGACCCAATGTGGAAAATCCC
VRF-CSRB-03	AGATAAATTATCTCCGATTTAAATCTTTTAAGGATGTTGGT

### Selected aptamers specifically bind to RB cells with high affinity

**Figure 3:**
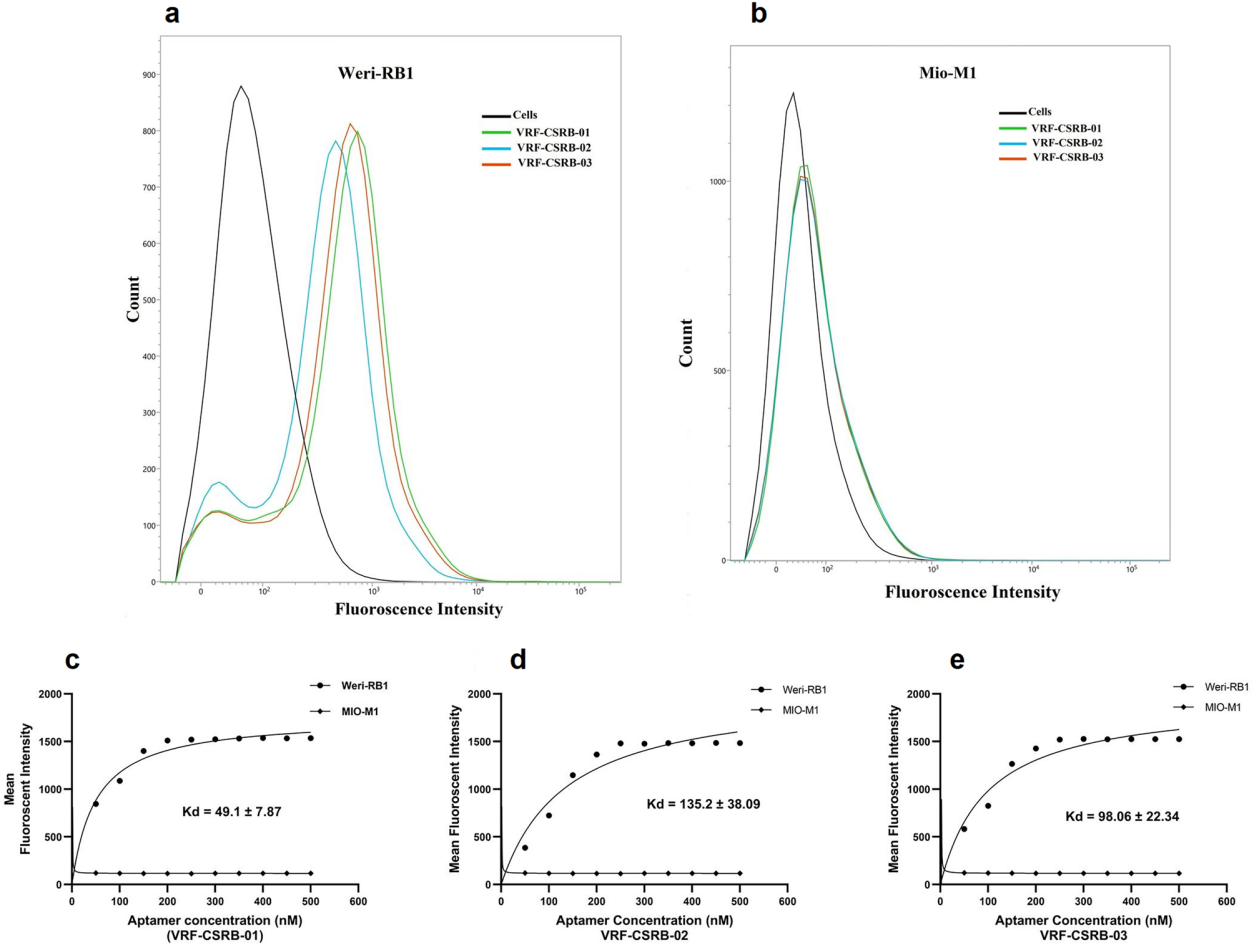
Binding ability and dissociation constants of selected aptamers. (**a**) Binding ability of FITC-labelled aptamers VRF-CSRB-01, VRF-CSRB-02 and VRF-CSRB-03 on Weri-RB1 cells assessed by flow cytometry. (**b**) Binding ability of FITC-labelled aptamers VRF-CSRB-01, VRF-CSRB-02 and VRF-CSRB-03 on Mio-M1 cells assessed by flow cytometry. (**c**–**e**) Binding curve of aptamers VRF-CSRB-01, VRF-CSRB-02, and VRF-CSRB-03 with Weri-RB1 and Mio-M1 cells. The cells were incubated with increasing concentrations of FITC-labelled aptamers and assessed by flow cytometry. Equilibrium dissociation constants (Kd) (nM) were calculated using GraphPad Prism 7, under the non-linear fit model, one-site non-competitive binding to fluorescent population ratio at used aptamer concentrations.

The binding assay of the selected aptamers on the target and control cells was performed by flow cytometry. The fluorescence shift of FITC-aptamers binding to Weri-RB1 cells demonstrated that all three aptamers bound to target cells with high affinity and had no specific binding to Mio-M1 cells. VRF-CSRB-01, VRF-CSRB-02, and VRF-CSRB-03 bound to approximately 94%, 88%, and 90% of the overall Weri-Rb1 population, respectively, with almost no positive signal detected for Mio-M1 cells (Fig. 3a,b).

Flow cytometry was performed to determine the affinity of the selected aptamer to Weri-RB1 and Mio-M1 cells to calculate Kd values. Figure 3c,d,e show the aptamers binding saturation curves with the target Weri-RB1 cell line, leading to Kd values of 49.41 ± 7.87 nM, 135.2 ± 38.09 nM and 98.06 ± 22.34 nM at 4 °C (48.93 ± 5.38 nM, 134.8 ± 98.69 nM and 97.84 ± 38.34 nM at 37 °C), respectively. In addition, saturation curves with the negative control Mio-M1 cell line are also shown. Aptamer VRF-CSRB-01, with a Kd value of 49.41 ± 7.87 nM, was stronger in binding than other aptamers and was chosen for characterization.

**Figure 4:**
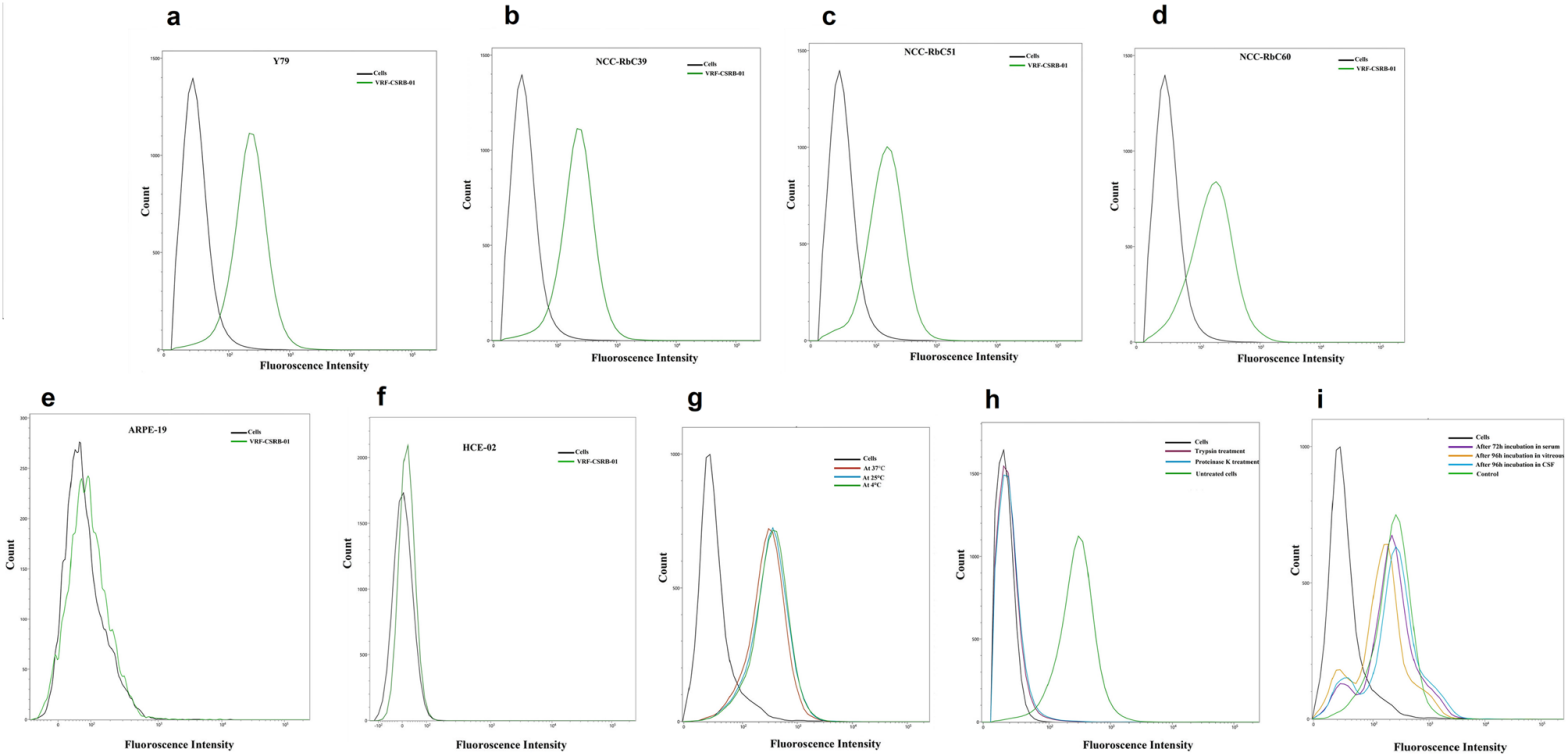
Binding ability and characterization of the selected aptamer VRF-CSRB-01. (**a**–**d**) The binding ability of VRF-CSRB-01 to RB cell lines Y79, NCC-RbC39, NCC-RbC51 and NCC-RbC60 respectively analysed by flow cytometry. (**e**) The binding ability of VRF-CSRB-01 to retinal pigment epithelial (ARPE-19) cell line. (**f**) The binding ability of VRF-CSRB-01 to corneal epithelium (HCE-02) cell line (**g**) Effect of incubation temperature on the binding ability of VRF-CSRB-01 with Weri-RB1 cells. The cells were incubated with FITC-labelled VRF-CSRB-01 at 4 °C, 25 °C and 37 °C and analysed by flow cytometry. (**h**) Binding ability of aptamer VRF-CSRB-01 with Weri-RB1 cells treated with trypsin or proteinase-K. The cells were treated with trypsin or proteinase K at 37 °C for 5 min and then incubated with FITC-labelled VRF-CSRB-01 aptamer and analysed by flow cytometry. (**i**) The binding ability of aptamer recovered from the biological fluid’s serum, vitreous and CSF after 72 and 96 h respectively.

The differential-specific binding of VRF-CSRB-01 against four other RB cell lines, Y79, NCC-RbC39, NCC-RbC51, and NCC-RbC60, showed a binding percentage of 88%, 86%, 89%, and 91%, respectively (Figs. 4a,b,c,d), was comparable to that of the Weri-RB1 and didn’t show any specific binding to ARPE-19 (Fig. 4e) and HCE-02 (Fig. 4f) cell lines, further validating that aptamer can recognize RB cells but no other cells related to the eye.

The fluorescence intensity patterns of Weri-RB1 cells incubated with VRF-CSRB-01 at different temperatures (25 and 37 °C) showed similar patterns and binding percentages to target cells (92% and 94%) as that of 4 °C, demonstrating that the incubation temperature has almost no effect on the binding capacity of the aptamer (Fig. 4g).

### VRF-CSRB-01 aptamer specifically targets cell surface receptors

To explore whether the VRF-CSRB-01 interacted with specific cell surface proteins on Weri-RB1 cells, protease K and trypsin digestion assays were performed. The flow-cytometry results show that the aptamers lost the binding ability to their target cells after treatment with either proteinase K or trypsin, suggesting the aptamers did bind to specific membrane proteins on target cells (Fig. 4h).

### The stability of chemically modified VRF-CSRB-01 aptamer in biological fluids

The stability of the VRF-CSRB-01 aptamer with or without phosphorothioate modification in serum, cerebro-spinal fluid (CSF), and vitreous was checked with Native PAGE (Fig S5) and the binding affinity of retrieved aptamers was evaluated by flow cytometry. The unmodified aptamer at 37 °C was stable in all biological fluids for 12 h, after which there was a degradation of the aptamer. In contrast, the modified aptamer at 37 °C was stable for 72 h in serum and 96 h in CSF and vitreous. The aptamer recovered from the biological fluids showed a similar binding affinity to target cells as the normal aptamer by flow cytometry (Fig. 4i).

### Targeted imaging for the translational potential of the aptamer

**Figure 5:**
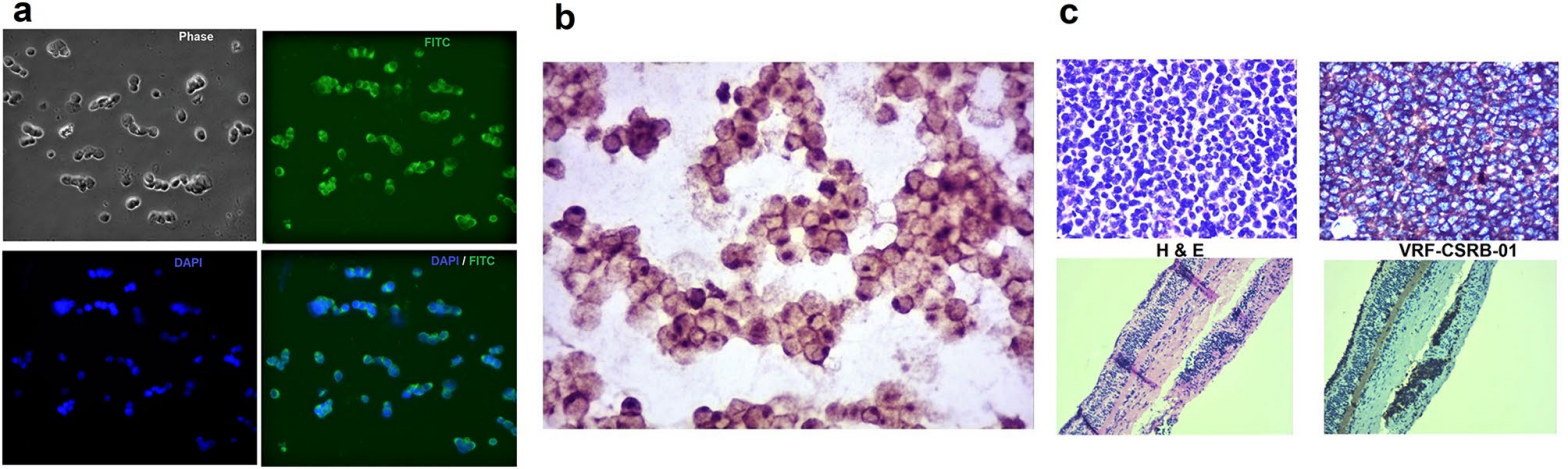
The binding ability of VRF-CSRB-01 to Weri-RB1 by IFC, ICC and RB tumour sections by IHC. (**a**) Microscopy results showed membrane positivity of VRF-CSRB01 by the fluorescence imaging in Weri-RB1. (**b**) ICC of Weri-RB1 cells incubated with biotin labelled VRF-CSRB-01 and analysed by light microscope. (**c**) H and E staining and () IHC with biotin labelled VRF-CSRB-01 of primary retinoblastoma tumours and retina respectively.

Aptamer VRF-CSRB-01, with a lower Kd value, was a slightly stronger binder than other aptamers and was chosen for tumour imaging. The apta-cytochemistry with FITC labelled aptamer (Fig. 5a) showed a visible green fluorescence signal, and biotinylated aptamer (Fig. 5b) showed specific binding to Weri-RB1 cells with strong membrane positivity.

To examine the feasibility of clinicopathological diagnosis, the staining of RB tumour sections with biotin-labelled aptamer showed significant binding with membrane positivity of the tumour but not the retina (Fig. 5c) or surrounding eye tissues (Fig S6), reconfirming the ability of the aptamer specificity to the tumour cells.

## Discussion

Retinoblastoma (RB) is a paediatric malignant eye tumour. The management of high-risk advanced RB and recurrence is challenging due to retinal toxicity and multi-drug resistance. The current therapeutic agents, are not functionalized to target the site selectively and lead to nonspecific biodistribution to nontargeted sites, which results in a reduced therapeutic index. Alternate modalities that selectively target RB, while sparing the normal retina can improve diagnosis, prognosis, and treatment outcomes. Aptamers are versatile probes that have potential both for diagnosis and treatment as they show high affinity and specificity for their targets^17^. While there are some aptamers designed against specific proteins over-expressed in cancers used in RB, there is still a lack of RB-specific aptamers that bind multiple types of RB cells and different grades of RB without using specific markers. To evolve aptamers that can recognise the RB tumour cells from other normal retinal cells without a prior selection of epitopes or targets, we adapted the whole Cell-SELEX system for use on Weri-RB1 cells. Using this approach, we identified and independently validated VRF-CSRB-01 aptamers, which have high affinity for proteins or cell surface antigens that are specific to and present on the surface of RB cells, but not on control cells.

The key to developing molecular probes for therapeutic application lies in reducing the side-effects of non-specific targeting. Our findings confirmed that the aptamer VRF-CSRB-01 had higher affinity and sensitivity for RB cell lines established from both primary site (Weri-RB1, Y79, NCC-RbC39, and NCC-RbC60), and metastatic sites (NCC-RbC51) with 86% to 91% binding, while other aptamers (NCL and EpCAM) used for targeting RB (Weri-RB1 and Y79) showed only 40–60% of abundance in RB^32,33^, which are present in lower expression in normal non-cancerous tissues. Further, aptamer VRF-CSRB-01 didn’t bind to most common retinal (Mio-M1, ARPE-19) and ocular cell lines (HCE-02). To the best of our knowledge, VRF-CSRB-01 is the first ssDNA aptamer that has high abundance and binds to a wide array of RB cell types with negligible binding to control cells, demonstrating its specific potential for targeting different RB cell types.

Aptamers are susceptible to nuclease degradation, and have poor serum stability, especially RNA aptamers. So, we have used an ssDNA library as the initial pool for the aptamer development and modified the VRF-CSRB-01 aptamer with a phosphorothioate backbone. Most of the aptamer stability studies have been checked in serum for therapeutic applications. We studied and found that the phosphorothioate modification has increased the stability of the VRF-CSRB-01 aptamer not only in the serum, but even in RB-related biological fluids, such as vitreous and CSF. Further, the aptamer retained its potential to bind RB cells after recovery from the biological fluids. With such stability and sensitivity, this aptamer can be conjugated to the gold nanorod for the enhancement of the signal or to gold nanoparticles or quantum dots for use in colorimetric or fluorescence detection of RB cells in liquid biopsies.

The aptamers developed against specific proteins (NCL and EpCAM)^32,33^ whose expression in RB was predominantly validated by flow cytometry using aptamers and by IHC using antibodies, showed only < 60% cells with positive staining on average. The affinity of VRF-CSRB-01 for RB primary tumours was evaluated by IHC because the goal is to be able to use the aptamer clinically. The aptamer VRF-CSRB-01-based apta-histochemistry assay can specifically diagnose clinical RB cancer tissues of various histopathological with more than 90% membranous positivity. In addition, the apta-histochemistry didn’t show any background or non-specific binding to the retina or other eye tissues. And no matter whether aptamer is modified with fluorophore or biotin, its specific binding ability to RB cells is not changed. This may hint that this aptamer could be further conjugated with new types of luminescent or imaging materials and thus be developed into promising molecular probes for clinical pathological diagnosis and imaging of RB.

The VRF-CSRB-01 aptamer's target is an epitope on cell surface proteins, and its high expression in primary tumours (> 90%), stability, and ability to bind at various physiological temperatures (4 °C, 25 °C, and 37 °C) make it a promising probe for conjugating the aptamer with suitable chemotherapeutic agents, siRNA, miRNA, and pri miRNA, among others, for efficient tumour cell targeting.

## Conclusion

In summary, we have developed a DNA aptamer that can bind to RB cells and primary tumours with high specificity and affinity. The selected aptamer obtained after 26 rounds was characterized regarding its binding affinity to the target, with a Kd value 49.41 ± 7.87 nM and was found to be associated with cell membrane protein of target cells. The potential translational application was proven for VRF-CSRB-01 by stability in the biological fluids and immunohistochemistry staining of RB tissue sections. The selected aptamer can be conjugated with therapeutic agents, including small interfering RNAs (siRNAs), CRISPR-Cas9, and chemotherapeutic drugs to enhance therapeutic efficacy and reduce associated toxicities. Conjugating the aptamer with chemotherapeutic drugs, would be actively delivered to the RB cells with better penetration and reduce side effects than free drugs. Further studies will focus on identifying the receptor of this aptamer and the effectiveness of the functionality of this aptamer for targeted delivery.

## Methods

### Ethics statement

The study was conducted in compliance with the declaration of Helsinki, and approved by the Institutional Ethics committee of the Vision Research Foundation, (Ethics Number: 405–2014-P) Chennai, Tamil Nadu, India.

### Cell lines and buffers

Retinoblastoma cell-lines, Weri-RB1, Y79, NCC-RbC-39, NCC-RbC-51, NCC-RbC-60 and retinal pigment epithelial cell line (ARPE-19) were purchased from Riken and human corneal epithelial cell line (HCE-02) from ATCC. A non-cancerous Muller glial cell-line, Mio-M1 was derived from the neural retina and was a gift from Dr. Professor Astrid Limb (UCL, Institute of Ophthalmology, London, England). Retinoblastoma cell-lines were cultured in RPMI medium, Mio-M1, were cultured in DMEM, ARPE-19 in DMEM-F12 and HCE-02 in keratinocyte-serum free medium supplemented with 5 ng/ml of EGF (epidermal growth factor), 500 ng/ml hydrocortisone and 0.005 mg/ml insulin. All the cell-culture media are supplemented with 10% FBS, 100 U penicillin/ml, 100 μg streptomycin/ml and 0.25 μg amphotericin B/ml and cells were cultured in a humidified 5% CO_2_ incubator at 37 °C. Cell-culture media and supplements are from Gibco (Thermo-Fisher Scientific, IN). All experiments were done with 80% confluent cultures within 2–4 passages.

### ssDNA Library and Primers

An initial ssDNA aptamer library containing 40 bases (5’-AGGGAAGAGAAGGACATATGAT–40 N–TTGACTAGTACATGACCACTTGA-3’) randomized sequences were obtained from Trilink Biotechnologies, San Diego, US. The FITC-labelled forward primer (5’-TAGGGAAGAGAAGGACATATGAT-3’) and a biotinylated reverse primer (5’-TCAAGTGGTCATGTACTAGTCAA -3’) were used in PCR for the synthesis of dsDNA. The primers and aptamers were synthesized by Integrated DNA Technologies (IDT).

### SELEX process

The Cell-SELEX procedure is as previously described by Shangguan et al. 2006^49^ with minor modifications. In this study, Weri-RB1 was used as the target cell line and the Mio-M1 cell line was used for counter-selection. Weri-RB1 and Mio-M1 cells were grown to 80% confluence in 60 mm culture dishes. Prior to each round, the native ssDNA library dissolved in Binding Buffer [BB–Wash Buffer with 0.1 mg/mL yeast tRNA (Invitrogen), 0.1 mg/mL Salmon sperm DNA (Ambion, Life technologies), 5% FBS (Gibco) and 1 mg/ml BSA (Sigma)] was denatured by heating at 95 °C for 5 min, snap-cooled on ice for 3 min, to reduce intermolecular hybridization and allow each DNA sequence to form the most stable secondary structure. For the first four rounds, 1 × 10^7^ Weri-RB1 cells were rinsed with Wash Buffer [WB–25 mM Glucose (Sigma), 0.9 mM CaCl_2_, and 5 mM MgCl_2_ in Dulbecco’s PBS without calcium and magnesium (Gibco)] twice after PBS wash and incubated on ice with 20 nmol of aptamer library dissolved in BB for two hours with gentle agitation. After incubation, cells were collected and centrifuged at 1000 rpm for 3 min at 4 °C. The sequences bound to Weri-RB1 cells were recovered by heating at 95 °C for 15 min, cooled on ice for 5 min, and eluted into 500 μl of deionized water by centrifugation at 14,000 rpm for 5 min. The recovered sequences are used as templates and then amplified by PCR with the primers modified by FITC and biotin (Initial denaturation at 95 °C for 5 min, 16 cycles of 30-s denature at 95 °C, 30-s annealing at 59 °C, and 30-s extension at 72 °C, the final extension by 5 min at 72 °C). The strand separation of the PCR product was performed by streptavidin-coated sepharose beads (GE Healthcare, USA) followed by desalting with a NAP-5 column (GE Healthcare); the generated FITC-labelled ssDNA pool was lyophilized and used as a pool for following the round of selection.

Counter-selection was introduced with Mio-M1 cells from the fifth round and subsequent selection cycles. Briefly, 1 × 10^6^ Mio-M1 cells were incubated with the selected ssDNA pool at 4 °C for 30 min with gentle agitation. The unbound ssDNA sequences were then separated by centrifugation at 14,000 rpm for 5 min and subjected to further positive selection. The selection stringency was progressively enhanced to select aptamers that specifically recognize Weri-RB1 cells (Table 2).

**Table 2:** Increasing the stringency of selection conditions in cell-SELEX process.

Cycle	Cells number	ssDNA (nmoles)	Yeast tRNA /Salmon sperm DNA (µg/ml)	Incubation time (Min)	No of washes (min)
1	1 × 10^7^	20	0.1/0.1	120	1 × 1
4	0.5 × 10^7^	20	0.1/0.5	120	1 × 3
8	0.25 × 10^7^	16	0.5/0.5	80	2 × 2
12	1 × 10^6^	16	0.5/1	80	2 × 3
16	0.5 × 10^6^	12	0.5/1	60	3 × 1
20	0.25 × 10^6^	10	1/1	60	3 × 2
24	1 × 10^5^	10	1/1	30	3 × 3
28	0.5 × 10^5^	10	1/1	30	3 × 3

To monitor the enrichment during Cell-SELEX, 500 nM of FITC-labelled ssDNA pools were incubated with the 2 × 10^5^ Weri-RB1 and Mio-M1 cells (dissociated with non-enzymatic dissociation buffer) in 500 μL of BB at 4 °C for 30 min after washing once with 1X PBS and twice with WB. After incubating the enriched pools, the cells were washed three times with WB and analysed by flow cytometry (BD FACS Accuri). The FITC-labelled ssDNA library was used as a negative control. All experiments were repeated three times. A total of 30 rounds of selection were performed before sequencing the pools. After confirming the saturation of enrichment by FACS, the enriched pools were sent to Base Pair Biotechnologies (Texas, US) for Next-Generation Sequencing. The top 20 oligonucleotide sequences were further evaluated by phylogeny and the number of repeats in each family. The Geneious software was used to construct a phylogenetic tree with the Tree Builder function. The tree distances and sequence relatedness were determined by the method of Tamura and Nei using a neighbour-joining model without any outgroup. The secondary structure and free energy (Delta-G) of shortlisted aptamers were calculated using the M-fold web server (http://www.unafold.org/mfold/applications/dna-folding-form.php), finding the optimal structure for each aptamer. The conditions used for the structure predictions were set according to the constituents of the BB at 37 °C in 200 mM Na^+^ and 0.5 mM Mg^2+^ at pH 7.4. Sequence homology analysis by MEME online software (https://meme-suite.org/meme/tools/meme) to find out the homology between the sequences and identify the possible binding motifs.

### Binding assays

To evaluate the binding efficiency of selected aptamers, 250 nM of FITC-labelled aptamers were incubated with 5 × 10^5^ Weri-RB1 and Mio-M1 cells on ice for 30 min. Samples were washed three times with 0.5 mL of WB, resuspended in 0.5 mL of 1X PBS or BB and analysed by the BD FACS Lyric cytometer. The initial FITC-labelled ssDNA library and unstained cells served as controls.

To determine the equilibrium dissociation constant 5 × 10^5^ Weri-RB1 and Mio-M1 cells were incubated with varying concentrations (0 to 500 nM) of FITCl-abelled aptamers in 200 μL of BB on ice for 60 min in the dark with gentle shaking. Cells were then washed thrice with 0.5 mL of WB, re-suspended in 0.5 mL of BB, and subjected to flow cytometric analysis. The ssDNA library was used as the negative control. The mean fluorescence intensity of each sample was subtracted from the mean fluorescence intensity of the background produced by the ssDNA library. The equilibrium dissociation constant (Kd) of the aptamer–cell interaction was obtained using GraphPad Prism (GraphPad Prism version 7.00 for Windows) by fitting the dependence of fluorescence intensity of specific binding on the concentration of the aptamers to the equation:

$$ Y = Bmax \times X/\left( {Kd + X} \right) $$

where, Y is fluorescence intensity of cells, X is concentration of the aptamer and B_max_ is maximum binding potential.

To determine the specificity of the selected aptamer, the binding assay of VRF-CSRB-01 with four other RB cell lines (Y79, NCC-RbC39, NCC-RbC51, and NCC-RbC60), and normal ocular cell lines, retinal pigmental epithelial cell line (ARPE-19) and corneal epithelial cell line (HCE-02) was analysed by flow-cytometry.

To check the effect of temperature on the binding affinity, Weri-RB1 cells were incubated with 250 nM VRF-CSRB-01 at 25 °C and 37 °C for 30 min, while aptamers incubated on ice were used as the positive control and the ssDNA library as a negative control, followed by flow-cytometric analysis.

To perform a target-type analysis, Weri-RB1 cells were treated with a 0.1 mg/mL Proteinase K (Sigma) or 0.25% trypsin (Gibco) solution for 5 min at 37 °C. The reaction was stopped by adding 5 ml of RPMI with 20% (v/v) of FBS. Then, cells were washed twice and incubated with 250 nM of VRF-CSRB-01 for 30 min and analysed by flow-cytometry.

### Stability of chemically modified aptamer in biological fluids

To assess the stability, 3 μM of unmodified and phosphorothioate aptamer was incubated for different time intervals (12, 24, 48, 72, 96, and 120 h) at 37 °C in various biological fluids like the serum, cerebrospinal fluid, and vitreous relevant to RB. After incubation for the assigned time interval, a 50 μl sample was removed and immediately placed in a −80 °C freezer for storage until all samples were harvested. Each sample was electrophoresed on a 12% native PAGE and checked using a Bio-rad gel imaging system. Three independent experiments were performed. To check if the aptamer retains its binding affinity with the target cells, the aptamer was recovered by proteinase-K digestion followed by ethanol precipitation from the biological fluids and checked for the binding affinity with the Weri-RB1 cells by flow-cytometry with the normal aptamer as the positive control.

### Histological analysis

To investigate the feasibility of the obtained aptamer for clinical diagnosis, immunocytochemistry on Weri-RB1 cells using FITC labelled and biotinylated aptamer and immunohistochemistry on FFPE sections of RB were performed using biotinylated aptamer. For ICC, Weri-RB1 cells were washed thrice with PBS and a cell smear was prepared and fixed with 4% paraformaldehyde in PBS for 5 min at room temperature. After fixation, slides were washed twice with PBS and blocked with binding buffer for FITC aptamer and endogenous biotin-blocking (Molecular probes E-21390) followed by HRP blocker (Dako K8023) for biotinylated aptamer for 30 min and washed three times with PBS. Slides are probed with 250 nM of FITC and biotinylated aptamer for 30 min at room temperature and washed with PBS thrice. Slides incubated with FITC labelled aptamer were mounted with DAPI Fluoromount-G (Invitrogen 00–4959-52) and imaged by a Zeiss microscope. Cells incubated with biotinylate aptamer were probed with streptavidin HRP (Dako K0609) for 30 min at room temperature, washed and incubated with HRP plus (Dako K8023) for 15 min. After washing, the slides were stained with DAB for 5 min, counterstained with haematoxylin for 1 min and mounted and analysed under the light microscope (Nikon Eclipse Ci–L, Tokyo, Japan).

The FFPE sections of retrospective RB samples and non-RB donor were deparaffinized and antigen retrieval was done by standard procedure using 0.01 M citrate buffer (pH 6.0). Antigen-retrieved tumor sections were blocked and processed as in ICC of biotinylated aptamer. Haematoxylin and Eosin (H and E) staining of RB tumour sections was performed.

## Supplementary Information

Supplementary Information.

## Data Availability

Data and aptamer sequences were uploaded at NCBI and can be downloaded using the accession number PRJNA844177.
