# Supplementary material for: Development and characterization of DNA aptamer against Retinoblastoma by Cell-SELEX
Source: Sci Rep. 2022 Sep 28;12:16178. doi: 10.1038/s41598-022-20660-3 (PMC9519959; doi:10.1038/s41598-022-20660-3)
Supplement: Supplementary file 1 — Supplementary Information. [file 41598_2022_20660_MOESM1_ESM.pdf]

Development and characterization of DNA aptamer against Retinoblastoma by Cell-SELEX

Bhavani Shankar Maradani[1,3], Sowmya Parameswaran[2], Krishnakumar Subramanian[1*]

*Corresponding author: drkk@snmail.org, drkrishnakumar_2000@yahoo.com

Running title: Retinoblastoma specific aptamers

Processing of the NGS data:

The quality of fastq files was assessed using FastQC software v0.11.2 (http://www.bioinformatics.babraham.ac.uk/projects/fastqc/). Adapters and primers were removed using Cutadapt v1.9.1 (https://cutadapt.readthedocs.io/en/stable/index.html) (Marcel, 2011). The paired ends were merged using Flash software v1.2.11 (https://ccb.jhu.edu/software/FLASH/) (Magoc and Salzberg, 2011) and the quality filtering was performed by Fastx toolkit (http://hannonlab.cshl.edu/fastx_toolkit/index.html). Sequences with more than 50% of bases of quality score below 30 were discarded. Trimmed sequences shorter than 38 and longer than 42 nucleotides were discarded as the library is of N40, leaving only the variable region sequences for downstream analysis. Aptamers were further shortlisted by AptaSUITE (Honika et al 2018).

Table S1: The sequencing parameters, demonstrating that the number of sequences analysed for the total experiment was in the millions.

**Parameter**	**Value**
Total Processed reads	68356492
Contig assembly failure	0
Total accepted reads	64896453
Invalid bases	109875
5' Primer error	249876
3' Primer error	214628
Total primer overlaps	42

Table S2: Frequency of the top aptamers in the sequenced pools.

**Aptamer**	**8 - Positive**	**12 - Positive**	**16 - Positive**	**22 - Positive**	**26 - Positive**
VRF-CSRB-01	4.80E+07	1.26E+08	2.80E+08	4E+08	6E+08
VRF-CSRB-02	3.60E+07	1.42E+08	2.38E+08	3.26E+08	5.2E+08
VRF-CSRB-03	4.20E+07	1.34E+08	2.26E+08	3.42E+08	5.02E+08
VRF-CSRB-04	3.80E+07	1.3E+08	2.20E+08	3.54E+08	5.12E+08
VRF-CSRB-05	3.40E+07	1.32E+08	2.16E+08	3.45E+08	5.18E+08
VRF-CSRB-06	3.20E+07	1.32E+08	2.16E+08	3.38E+08	5.06E+08
VRF-CSRB-07	3.00E+07	1.3E+08	2.10E+08	3.4E+08	4.98E+08
VRF-CSRB-08	2.60E+07	1.27E+08	2.04E+08	3.28E+08	4.96E+08
VRF-CSRB-09	2.40E+07	1.24E+08	2.00E+08	3.36E+08	4.8E+08
VRF-CSRB-10	2.40E+07	1.22E+08	1.98E+08	3.16E+08	4.82E+08
VRF-CSRB-11	2.10E+07	1.22E+08	1.98E+08	3.18E+08	4.76E+08
VRF-CSRB-12	2.00E+07	1.24E+08	1.96E+08	3.26E+08	4.78E+08
VRF-CSRB-13	2.10E+07	1.25E+08	1.98E+08	3.1E+08	4.6E+08
VRF-CSRB-14	1.80E+07	1.22E+08	2.00E+08	3.08E+08	4.52E+08
VRF-CSRB-15	1.60E+07	1.2E+08	2.02E+08	3.09E+08	4.58E+08
VRF-CSRB-16	1.50E+07	1.16E+08	1.97E+08	3.06E+08	4.62E+08
VRF-CSRB-17	1.70E+07	1.12E+08	1.96E+08	3.05E+08	4.5E+08
VRF-CSRB-18	1.40E+07	1.1E+08	1.92E+08	3.05E+08	4.42E+08

VRF-CSRB-19	1.30E+07	1.1E+08	1.92E+08	3.02E+08	4.4E+08
VRF-CSRB-20	1.20E+07	1.08E+08	1.92E+08	3E+08	4.28E+08

Figure S1: Selection of candidate aptamers by AptaSUITE using the NGS data. The distribution of the reads per round is shown in panel A. The library started out, and maintained a relatively equal base distribution (panel B) throughout the selection, indicating that the library was not skewed towards a particular base.

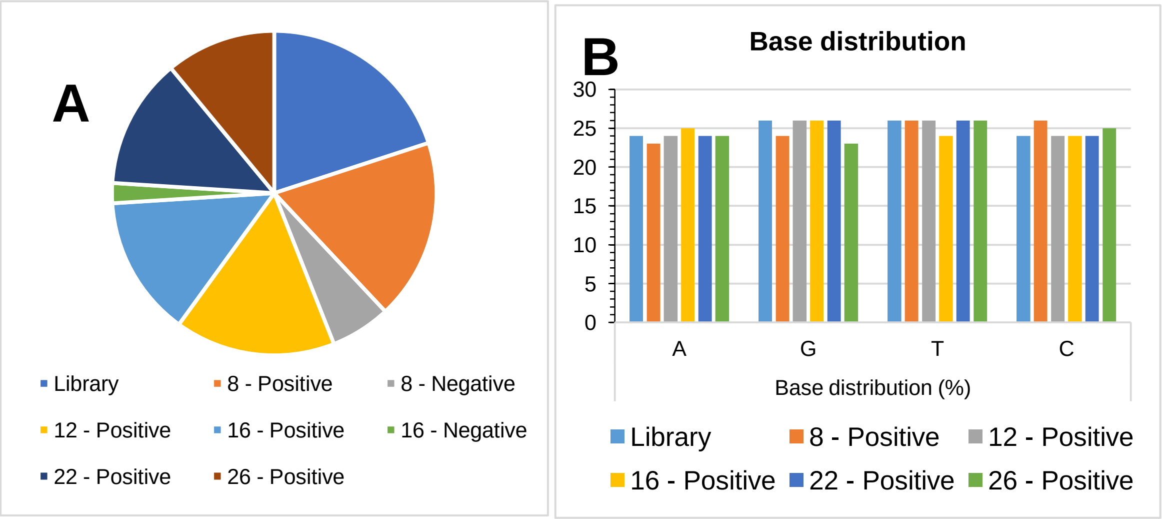

Figure S2: Enrichment of the sequences with the progression of the SELEX. Panel shows the fraction of the library and enriched pools that had singletons, unique sequences and enriched per selection round. The enriched pools have the most unique sequences .

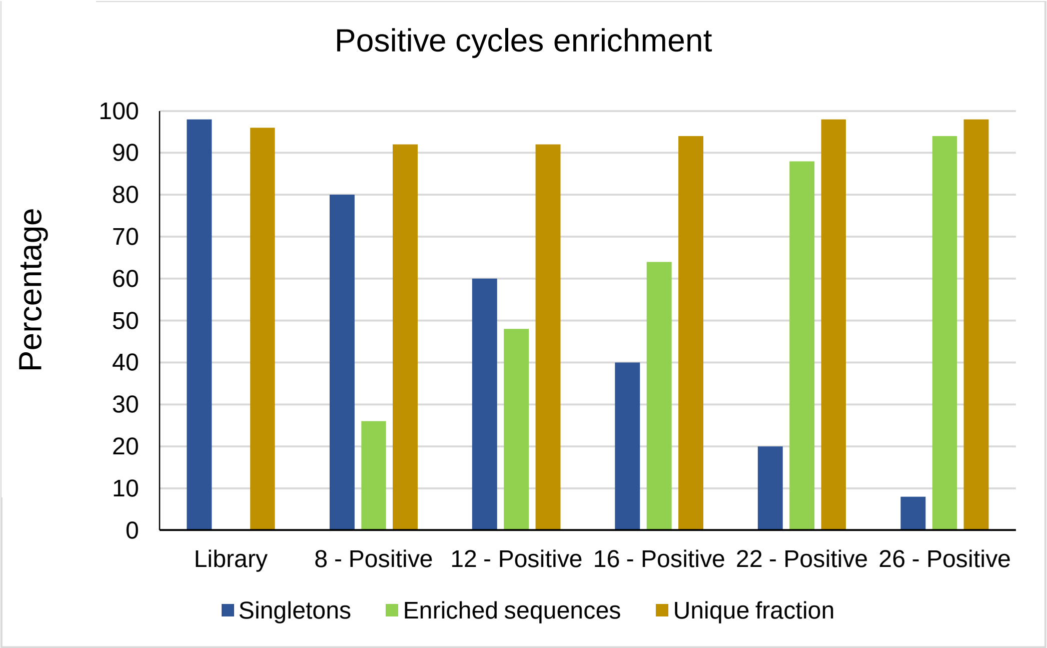

Figure S3. Secondary structures of top selected aptamers. Predicted secondary structures for the other seventeen aptamer candidates. The presented predicted secondary structures were the ones with lowest ΔG. Constant sequence regions are highlighted in black, and green represents the random regions.

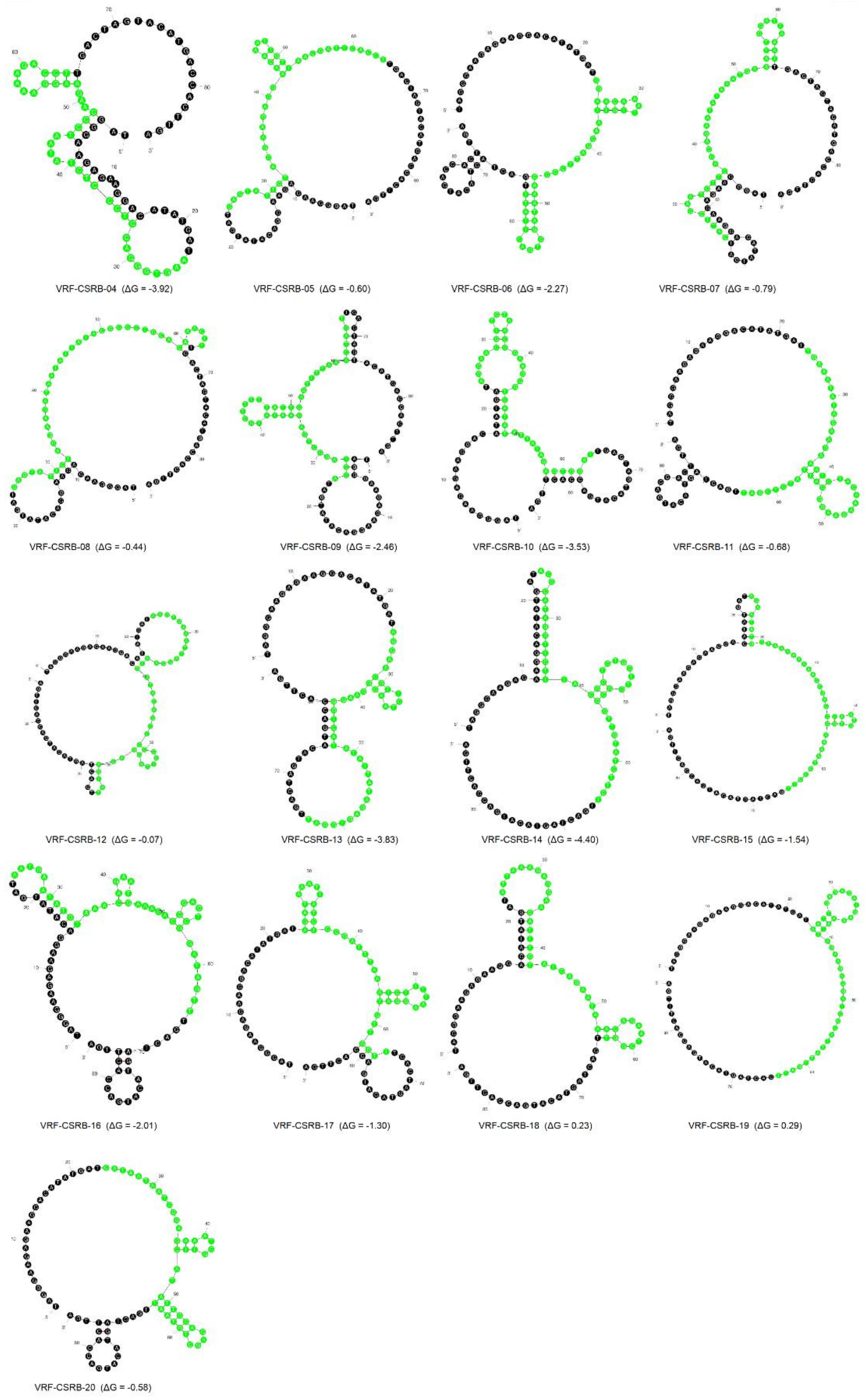

Figure S4: Summary of enrichment and count of the top three aptamers from the high throughput sequencing of the positive pools.

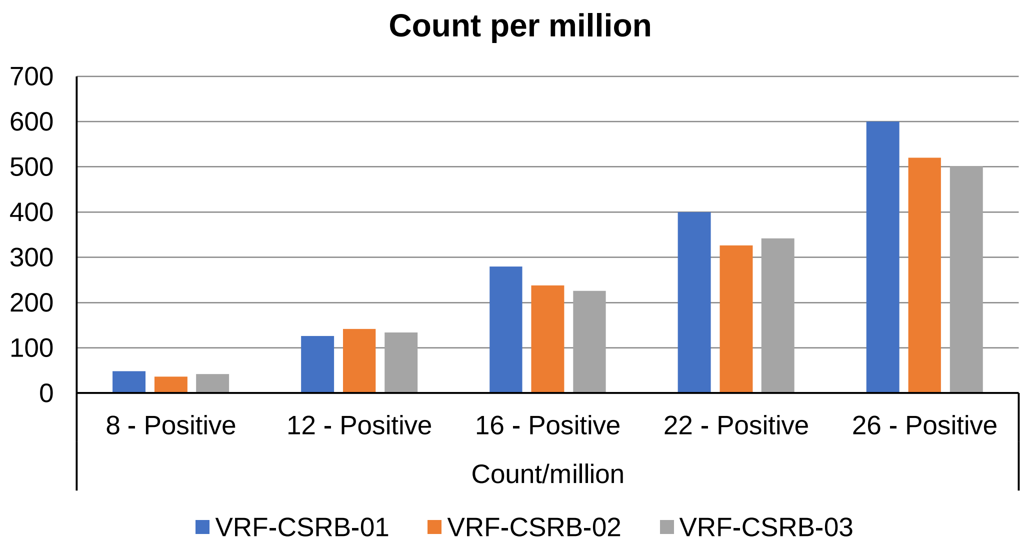

Figure S5: Stability of VRF-CSRB- with phosphorothioate modification in (a) serum, (b) CSF and (c) vitreous. C – control aptamer, 0-120 – time in minutes.

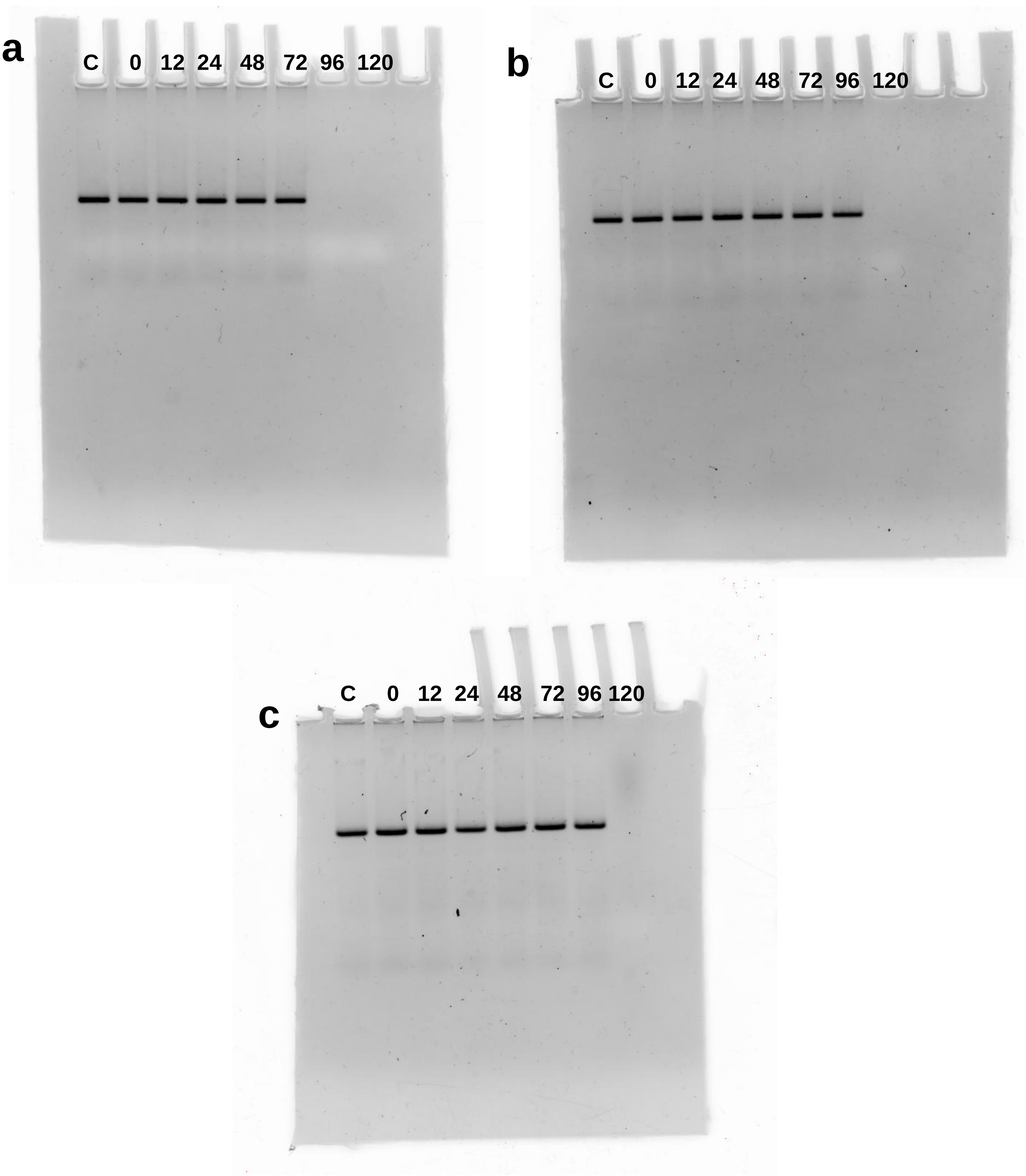

Figure S6: The binding affinity of VRF-CSRB-01 to different eye tissues by IHC. (a) IHC for VRF-CSRB01 on cadaveric non-RB eye ball and (b) enucleated painful eye ball revealed absence of binding of the aptamer. IHC for VRF-CSRB01 on enucleated retinoblastoma eye ball revealed non-binding of aptamer to various eye tissues other than RB tumor viz. (c) Corneal epithelium and corneal stroma, (d) Corneal stroma and corneal endothelium, (e) Lens epithelium, (f) Ciliary epithelium (negative) with vitreous seeds (positive), (g) Sclera with fibroblasts, (h) Choroid and RPE, (i) RB Tumor invading front of optic nerve, (j) optic nerve without tumor cells, and (k) Tumor cells in optic nerve.

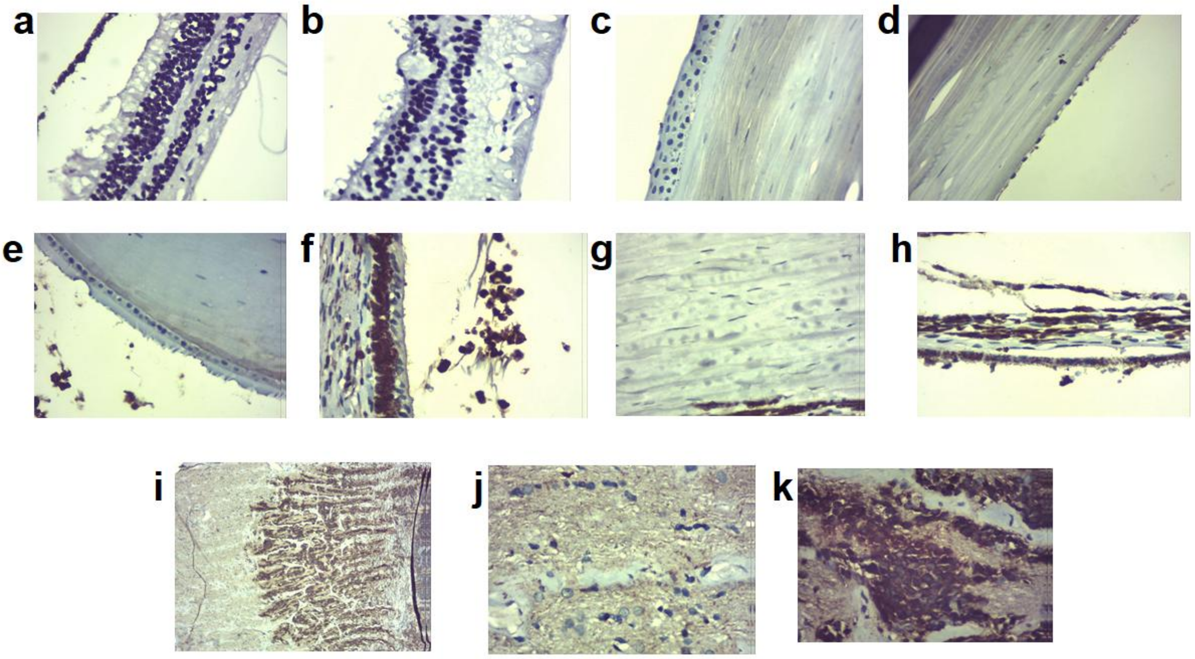

References:

Hoinka, J., Backofen, R., & Przytycka, T. M. AptaSUITE: A Full-Featured Bioinformatics Framework for the Comprehensive Analysis of Aptamers from HT-SELEX Experiments. *Molecular Therapy - Nucleic Acids*. **11**, 515–517 (2018).

Magoc, T., and Salzberg, S.L. FLASH: fast length adjustment of short reads to improve genome assemblies. *Bioinformatics*. 27, 2957-2963 (2011).

Marcel, M. Cutadapt removes adapter sequences from high-throughput sequencing reads. *EMBnet.* 10.14806/ej.17.1.200. (2011).
